# Magnetically Actuated Soft Electrodes for Multisite Bioelectrical Monitoring of Ex Vivo Tissues

**DOI:** 10.34133/cbsystems.0434

**Published:** 2025-10-24

**Authors:** Qianbi Peng, Jianping Huang, Chenyang Li, Mingguo Jiang, Chenyang Huang, Jinxin Luo, Hanfei Li, Ting Yin, Mingxue Cai, Shixiong Fu, Guoyao Ma, Zhiyuan Liu, Tiantian Xu

**Affiliations:** ^1^ Guangdong Provincial Key Lab of Robotics and Intelligent Systems, Shenzhen Institutes of Advanced Technology, Chinese Academy of Sciences, Shenzhen, China.; ^2^ University of Chinese Academy of Sciences, Beijing, China.; ^3^ Guangdong Provincial Key Laboratory of Multimodality Non-Invasive Brain-Computer Interfaces, Shenzhen Institutes of Advanced Technology, Chinese Academy of Sciences, Shenzhen 518055, China.; ^4^ School of Mechanical, Electrical & Information Engineering, Shandong University, Weihai 264209, China.; ^5^ WeiHai Research Institute of Industrial Technology of Shandong University, Weihai 264209, China.; ^6^ Research Center of Nano Technology and Application Engineering, The First Dongguan Affiliated Hospital, School of Pharmacy, Guangdong Medical University, Dongguan, Guangdong 523808, China.; ^7^ Fuwai Yunnan Hospital, Chinese Academy of Medical Sciences, Affiliated Cardiovascular Hospital of Kunming Medical University, Kunming, China.; ^8^ The Key Laboratory of Biomedical Imaging Science and System, Shenzhen Institutes of Advanced Technology, Chinese Academy of Sciences, Shenzhen 518055, China.; ^9^ Center for Neurocognition and Social Behavior, Artificial Intelligence Research Institute, Shenzhen University of Advanced Technology, Shenzhen 518107, China.; ^10^ Shenzhen Key Laboratory of Minimally Invasive Surgical Robotics and System, Shenzhen Institutes of Advanced Technology, Chinese Academy of Sciences, Shenzhen, China.

## Abstract

Multisite electrophysiological monitoring of ex vivo tissues and organ models is essential for basic research and drug toxicity evaluation. However, conventional microelectrode arrays with fixed positions and rigid structures are insufficient for dynamic, curved tissue surfaces. Here, we present a magnetically actuated soft electrode (MSE) with precise navigation, adaptive attachment, and high-fidelity signal acquisition. Operating in a “locate–adhere–record–detach” cycle, the MSE enabled continuous multisite detection on beating ex vivo tissues. In isolated rat heart experiments, the MSE demonstrated millimeter-level navigation accuracy, stable contact, and high signal-to-noise ratio (average 28 dB). By integrating magnetic locomotion with electrophysiological sensing, this work establishes a programmable, actively addressable platform for multisite electrophysiological monitoring of organ models, tissue slices, and engineered constructs, offering broad potential for cardiotoxicity screening and cardiovascular research.

## Introduction

Ex vivo cultured organoids, tissue slices, and isolated organs serve as physiologically relevant models that bridge the gap between in vitro cultures and in vivo animal studies [1–4]. These systems preserve key structural, electrophysiological, and biochemical properties of native tissues while allowing precise environmental control and accessibility for manipulation [5–8]. As such, they have become indispensable platforms for investigating mechanisms, modeling disease progression, and evaluating drug responses [9–12]. To fully capture the dynamic behavior of these models, real-time, site-resolved electrophysiological monitoring of them is critical for evaluating their functional status and dynamic responses [13]. However, conventional microelectrode arrays (MEAs) face 3 major limitations in such complex and dynamic environments: (a) their rigid structures cannot conform to moving curved tissues such as beating hearts, leading to unstable contact and signal distortion [14–16]; (b) fixed electrode layouts lack spatial addressability, making it difficult to evaluate multiple regions across time [11,17]; and (c) cable connections constrain operational flexibility, hindering large-scale and automated measurements in perfusion chambers or 3-dimensional (3D) cultures.

Advances in soft and stretchable electronics offer promising solutions. Several ultrathin patch-type sensors have demonstrated good mechanical compliance and biocompatibility, such as flexible fibrous electrodes for implantable biosensing applications [18–20], ultrapermeable epidermal electrophysiological electrode [21], highly stretchable polymeric MEAs for in vivo electrophysiological interfacing [22,23], temperature-calibrated sweat biosensors [24], and multifunctional acoustic sensors for wireless communication and environmental perception [25]. Yet, most of these devices are passively fixed in place and lack the capability for active repositioning, limiting their utility in dynamic, multi-site monitoring scenarios.

Meanwhile, magnetically actuated soft robots have shown unique advantages in noncontact actuation and bioenvironmental navigation [26–33]. Previous studies have demonstrated the ability of external magnetic fields to drive miniaturized devices with high degrees of freedom in complex biological settings [34], particularly for targeted drug delivery and tissue interaction [35–41]. However, the integration of magnetic actuation with high-fidelity electrophysiological sensing in a soft, flexible platform remains largely unexplored [42].

**Fig. 1.**
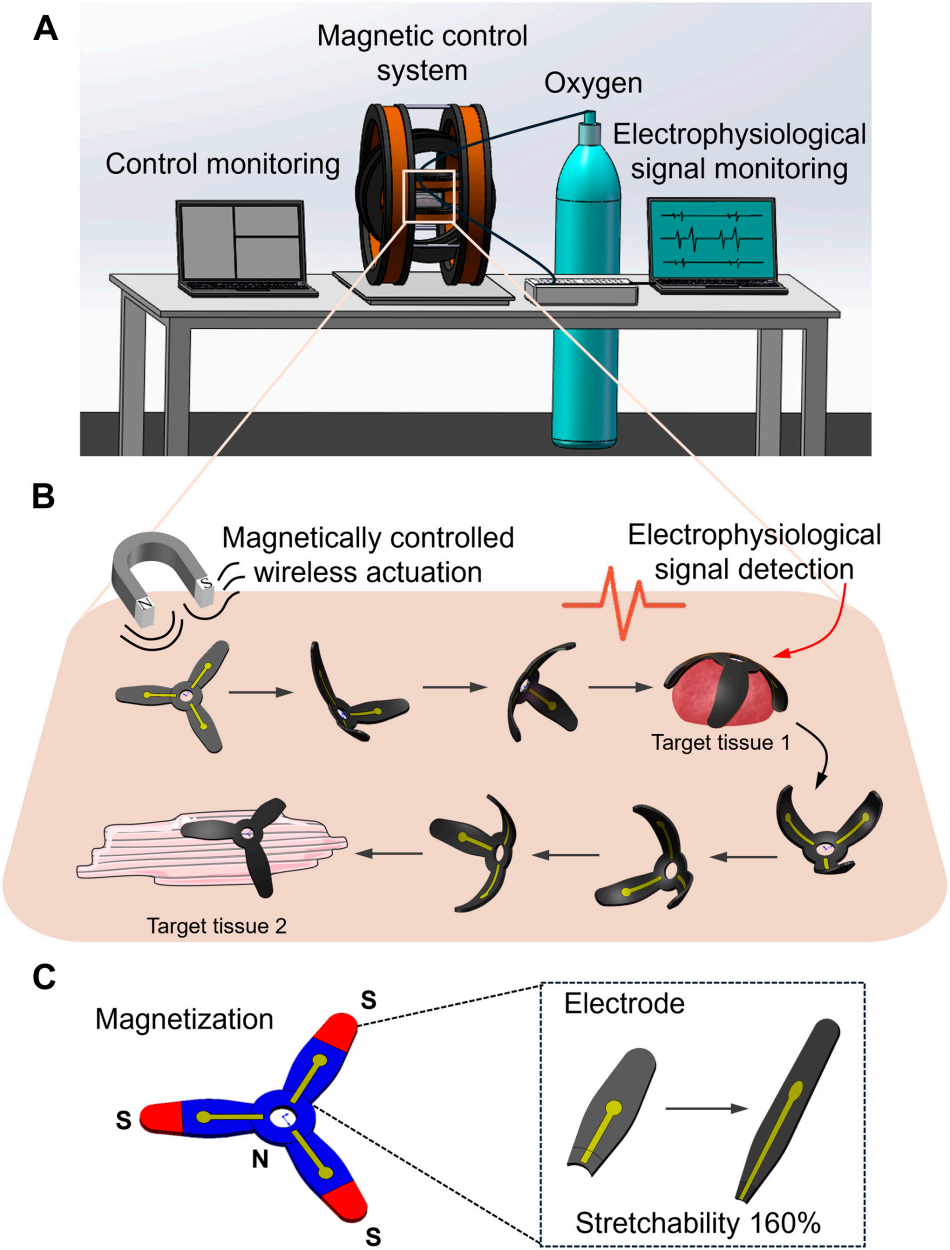
The diagram shows the concept of magnetically actuated soft electrode (MSE) for multisite electrophysiological monitoring of ex vivo tissues. (A) Schematic of the experimental setup. (B) MSE’s wireless magnetic actuation, tissue attachment, electrophysiological signal detection, and detachment cycle across multiple target sites. (C) Magnetization pattern, electrode distribution, and the stretchability of the MSE.

In this work, we present a magnetically actuated flower-shaped thin-film soft electrode (MSE; ~200 μm in thickness) designed for continuous multi-site electrophysiological monitoring of dynamic ex vivo tissues (Fig. 1). Structurally, the MSE features a compliant petal-like layout for stable contact with beating cardiac surfaces.

Functionally, it operates in a “locate–adhere–record–detach” cycle, allowing sequential recordings at multiple target tissues with a single device. Validation experiments on isolated beating rat hearts demonstrated precise navigation (<0.5 mm error), stable attachment, and high signal-to-noise ratio (SNR) (>28 dB) electrocardiograph (ECG) recordings. This is the first demonstration of integrating magnetic actuation and flexible electrophysiological sensing into a single platform, overcoming spatial constraints of fixed MEAs and enabling programmable, addressable multi-site monitoring. Unlike conventional passive MEAs, the MSE allows users to dynamically define measurement paths, such as scanning distinct functional zones in organoid assemblies. This flexibility offers new capabilities for studying conduction abnormalities, region-specific dysfunction, and spatially heterogeneous drug effects.

## Materials and Methods

### Experimental design

Fig. 1 illustrates the design and closed-loop operation of the MSE. The experimental setup includes a magnetic actuation system, an electrophysiological signal acquisition module, and an oxygen supply device (Fig. 1A). The operation takes place within a 10 cm × 10 cm × 5 cm workspace inside a 3D Helmholtz coil magnetic actuation system, where ex vivo tissues or organoids are placed in a culture dish. Under magnetic control, the MSE moves between multiple locations and records real-time electrical signals via connected wires with an electrophysiological signal acquisition module upon contact with tissues. The oxygen system delivered oxygen to the tissue every 5 min, with each cycle lasting about 20 s, to help maintain tissue viability.

The MSE operates in a closed-loop cycle (Fig. 1B): It is magnetically guided to the target tissue via a rotating field, adaptively adheres under a vertical constant field, records electrophysiological signals, detaches by removing the vertical constant field and reapplying the rotating field, and then moves to the next site. This enables repeatable, multi-site monitoring in a controllable manner. The MSE features a 3-petal structure and is magnetized under a 1-T field to form a tripolar distribution, with the petals and center magnetized as S and N poles (Fig. 1C). The resulting field strength reaches up to 2.7 mT. This magnetization enables controlled locomotion, targeted attachment, and detachment through the combined use of rotating and static magnetic fields (Movie S1). Furthermore, the electrode maintains its conductivity even under mechanical tensile strains of up to 160% (Fig. 1C), ensuring reliable signal recording from dynamic tissues such as a beating heart.

### Fabrication of MSE

**Fig. 2.**
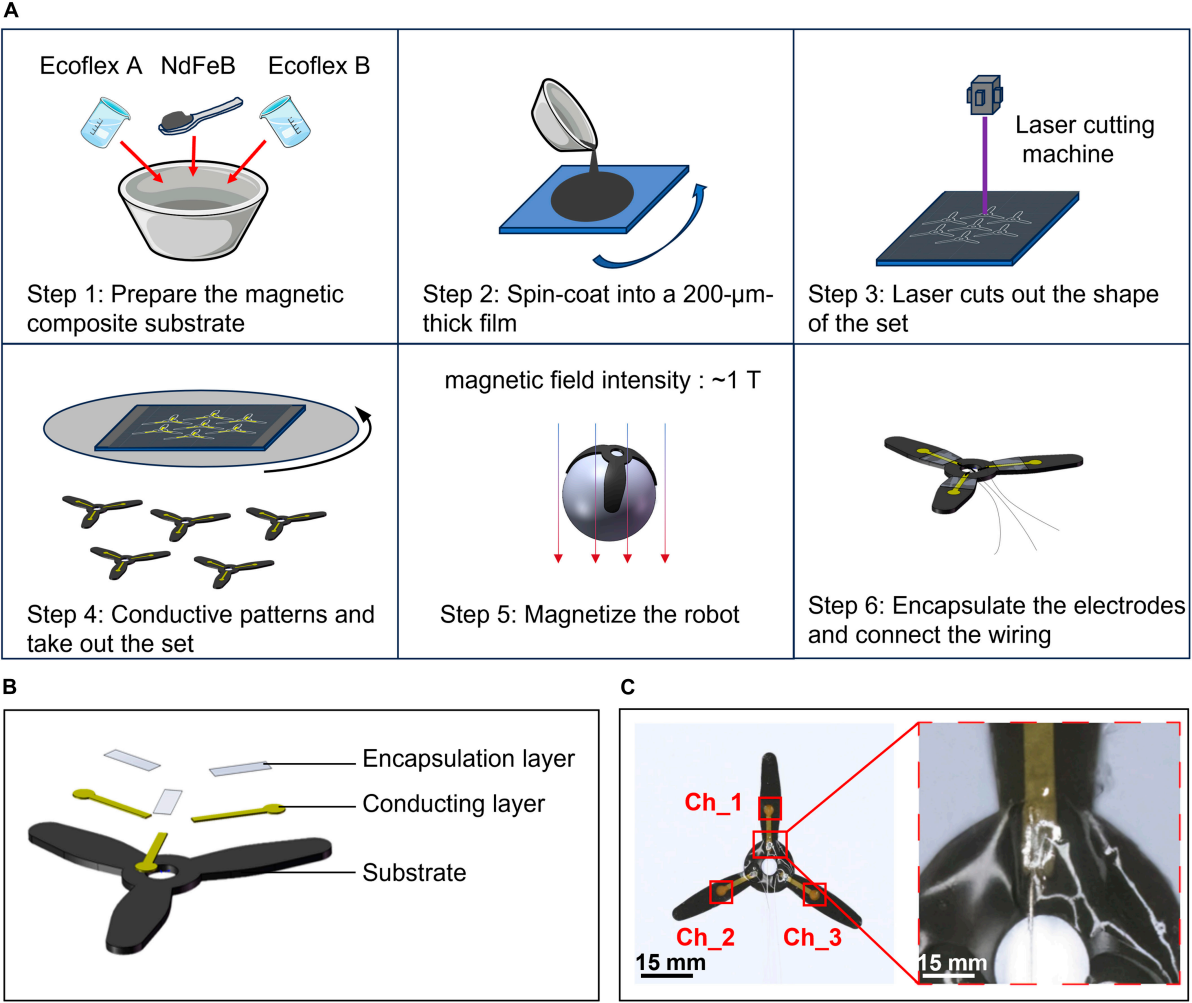
Fabrication process of MSE. (A) Ecoflex and NdFeB particles are mixed, poured onto glass, and spin-coated into a film. After laser cutting of the substrate, gold is deposited via thermal evaporation, followed by magnetizing and encapsulating to obtain the final MSE samples. (B) Schematic of MSE’s 3-layer structure. (C) Three petals corresponding to 3 channels, with a detailed view of the interface.

The MSE was fabricated using a multilayer casting and patterning process (Fig. 2A). The substrate layer consisted of the silicone elastomer (Ecoflex 00-30). To impart magnetic responsiveness, NdFeB magnetic microparticles (~5 μm) were dispersed into the silicone precursor at 40 wt % and spin-coated to a thickness of approximately 180 μm, followed by partial curing. The partially cured film was then laser-cut into a 3-petal flower shape (petal length: 15 mm; petal width: 4 mm). A patterned gold (Au) electrode layer (~500 nm) was subsequently deposited using a shadow mask and thermal evaporation. Magnetization was carried out using a pulsed magnetizer (Hunan Pai Sheng Technology Co., China) under a 1-T magnetic field. During magnetization, the device was wrapped around a 20-mm-diameter plastic rod to establish the tripolar magnetization pattern shown in Fig. 1C. At the final stage, the interface pads were connected to external wires via liquid metal to enable signal transmission. The 3-petal geometry was finalized after 5 iterative design optimizations. Key considerations included the small size of the rat heart (≈1 to 2 cm in diameter), the need for proximal lead routing, and the balance between petal contact area, magnetic actuation efficiency, and structural stability. The final design ensured reliable locomotion while maintaining sufficient electrode–tissue contact for high-quality signal acquisition.

Fig. 2B shows the 3-layer structure of the MSE. The substrate is made of an ultrasoft silicone elastomer with a Young’s modulus of approximately 50 kPa. A patterned thin Au film serves as the conductive layer on top of the substrate. The encapsulation layer, composed of pure silicone elastomer (~20 μm thick), covers the wiring region of the Au electrode pattern. The overall softness of the electrode enables it to conform seamlessly to the curved surfaces of dynamic biological tissues. Fig. 2C shows the 3 electrode channels of the MSE. Each electrode channel is 1 mm wide, and the sensing pads are 2 mm in diameter, providing both flexibility and stretchability.

The film thickness (200 μm) was selected as a balance between flexibility and robustness. Films thicker than 500 μm exhibited excessive stiffness and poor conformability, while those thinner than 100 μm suffered from curling, wrinkling, or insufficient magnetic responsiveness. Preliminary trials identified 200 μm as the optimal thickness to ensure tissue adaptability and reliable actuation.

### Statistical analysis

Magnetic actuation and motion control: All magnetic actuation experiments were conducted inside a 3-axis Helmholtz coil system capable of generating rotating or static magnetic fields up to 10 mT. The motion of the MSE was recorded by 2 cameras positioned above and beside the coil system. Step-out frequency and translational speed were characterized under varying magnetic field strengths (2 to 10 mT) and actuation frequencies (0 to 50 Hz).

Mechanical and electrical characterization: Tensile testing was performed on MSEs with a substrate width of 2 cm and electrode traces of 1 mm. The maximum tensile test was conducted at a strain rate of 1 mm/s. Cyclic stretching tests were performed on the same samples under 40% maximum strain for 4,000 cycles. The surface morphology of the electrode was examined using field-emission scanning electron microscopy (SEM), and surface roughness was measured using an optical profilometer.

Tissue adhesion and impedance measurements: To evaluate the adhesion of the MSE on biological tissue, the device was gently placed on the epicardial surface of freshly excised rat hearts. A uniaxial tensile test was performed using a mechanical testing system to measure interfacial adhesion force. A 1 cm × 1 cm electrode area was attached to the heart surface, and force was applied parallel to the film plane until complete detachment occurred. Impedance was measured using a CHI760e electrochemical workstation in a 3-electrode configuration over the frequency range of 1 to 10,000 Hz, with an effective sensing area of 1 mm^2^.

Ex vivo cardiac signal monitoring: All experiments were performed on male Sprague–Dawley (SD) rats weighing approximately 220 g at the time of testing. Freshly excised beating rat hearts were placed in a 10 cm × 10 cm × 5 cm culture dish filled with Krebs–Henseleit bicarbonate buffer. This buffer, continuously oxygenated with 95% O_2_ and 5% CO_2_, maintains a physiological pH of ~7.4 and is widely used in Langendorff isolated heart preparations to preserve both metabolic activity and electrophysiological integrity [43]. Oxygenation was refreshed approximately every 20 min to support tissue viability during the experiments. Beating duration varies across animals and depends on oxygenation, temperature, and handling; measurements were completed within the viable window after excision.

The dish was positioned within a 3D Helmholtz coil setup. For experiments involving 3 excised hearts in the same culture dish, the hearts were positioned at diagonal corners to maximize their separation. The MSE and its reference (REF)/ground (GND) electrodes were assigned to one heart at a time, ensuring that the acquired ECG signals originated specifically from that heart without interference from the others. Under a low-frequency rotating magnetic field (8 mT, 2.7 Hz), the MSE was sequentially navigated to 3 different heart surfaces. Upon reaching each target, the field was switched to a static vertical mode (8 mT) to achieve stable adhesion for signal acquisition. ECG signals were recorded at a sampling rate of 500 Hz and processed in MATLAB (R2022a, MathWorks, Natick, MA, USA). Signals were digitally filtered using a third-order Butterworth bandpass (0.5 to 100 Hz) to suppress baseline drift and high-frequency artifacts. The SNR was calculated as 20*log_10_(peak signal amplitude/RMS background noise), where RMS denotes the root mean square of the noise segment, computed as

$$\mathrm{RMS}=\sqrt{\frac{1}{n}\sum_{i=1}^{n}x_{i}^{2}}$$ (1)

where $x_{i}$ represents the individual sampled data points of the signal and n denotes the total number of data points considered. The built-in rms() function in MATLAB was used to ensure reproducibility.

## Results

### Magnetic actuation and locomotion

**Fig. 3.**
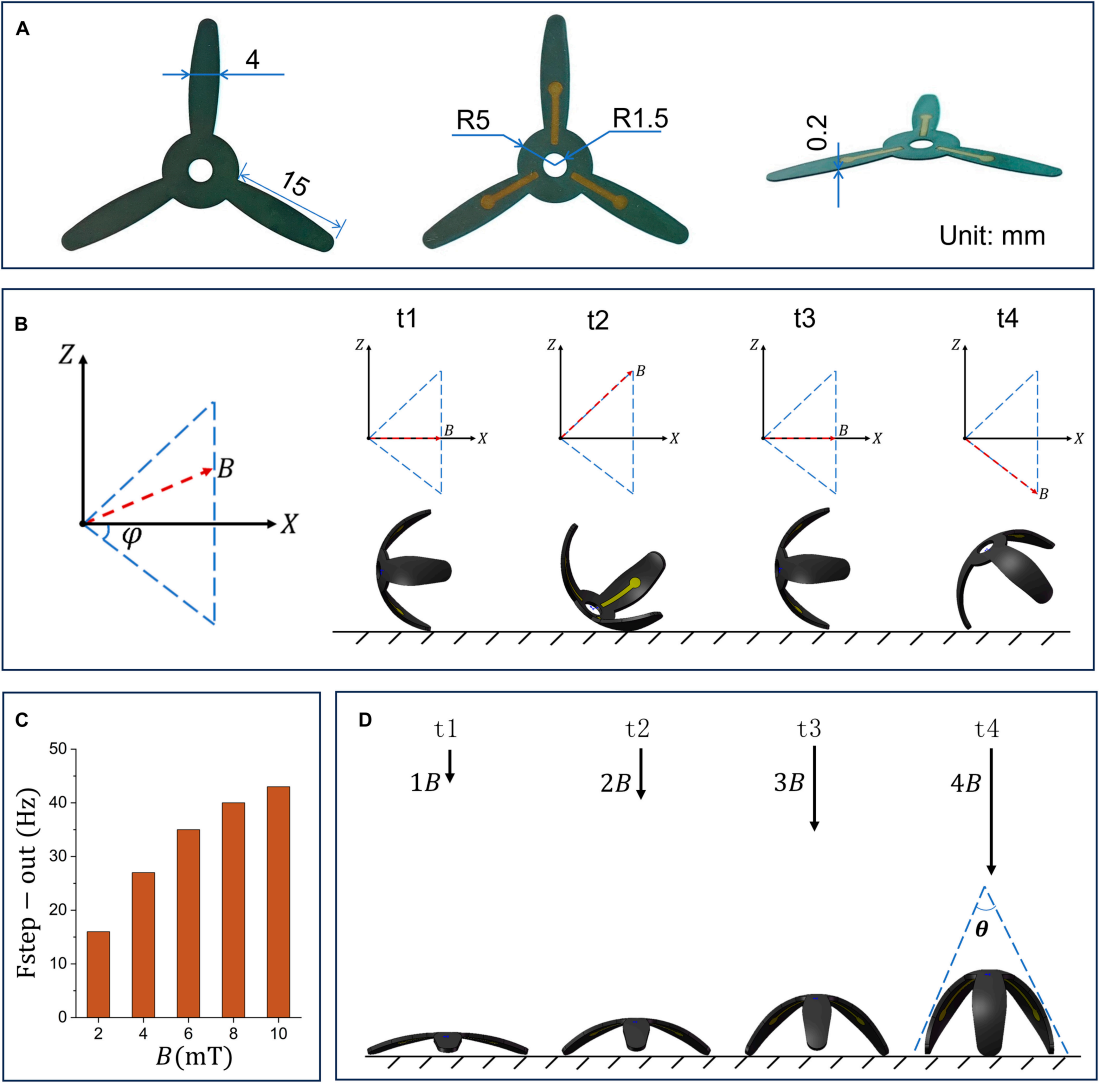
Magnetic actuation performance of MSE. (A) Structural design and dimensional parameters of the MSE, showing the back, front, and side views of the MSE from left to right. (B) Movement gait of MSE in a rotating magnetic field. (C) Step-out frequency of the MSE increases with magnetic field strength. (D) The angle of the MSE decreases as the magnetic field increases.

Figure 3 demonstrates the magnetic actuation performance of the MSE. The biomimetic 3-petal structure features a thin (~200 μm), lightweight body (~130 mg), enabling efficient magnetic-to-mechanical energy conversion (overall diameter: 35 mm; petal length: 15 mm; Fig. 3A). Under a rotating magnetic field, the MSE achieves programmable rolling locomotion with the ability to freely turn, move forward, or move backward (Fig. 3B). The rotating magnetic field was applied in the vertical ZOX plane, corresponding to the sagittal plane of the robot relative to the substrate. The rotating field induces a cyclic motion resembling the gait of a forklift shovel, during which the electrode petals sequentially lift, advance, and bow to generate forward locomotion between tissues. The rotation angle was determined geometrically to ensure completion of a full displacement cycle under the rotating field. Specifically, the angular range was set to *φ* ≈ 36.9°, derived from the relation 2·tan*φ* = 1.5. This angle was identified as optimal because it enables the robot to achieve efficient forward locomotion while minimizing excessive deformation. Fig. 3C presents the experimentally measured step-out frequency (*f*_step-out_), which increases linearly with magnetic field strength: As the field increases from 2 to 10 mT, *f*_step-out_ rises from 16 to 43 Hz, consistent with the theoretical relationship *f*_step-out_ ∝ *B* [44]. Here, the step-out frequency is defined as the maximum rotation frequency of the external field at which the MSE remains synchronized with it; above this, the MSE stops moving. Fig. 3D shows the precise magnetic control of the MSE’s orientation angle θ, which decreases as field strength increases, reaching a minimum at 8 mT.

**Fig. 4.**
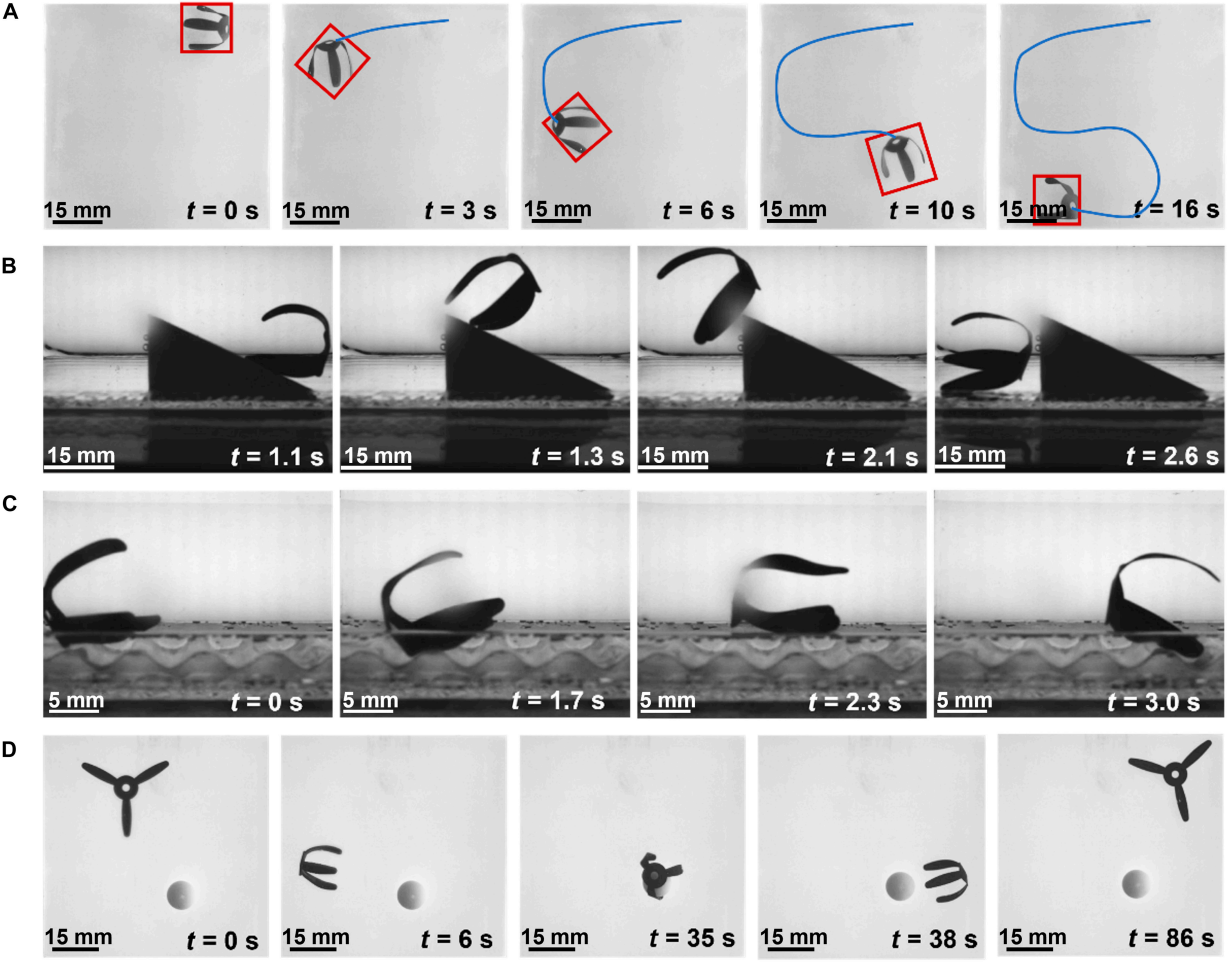
Video snapshots of different locomotion modes of MSE. (A) MSE follows an S-shaped path. (B) MSE climbs a 25° slope. (C) MSE walks on rough terrain. (D) MSE reorients, approaches, attaches to, and detaches from a target (*B* = 8 mT, *f* = 22 Hz).

Fig. 4 shows snapshots of the MSE in different locomotion modes (see Movie S1). Fig. 4A to C demonstrates the MSE moving along an S-shaped path (8.5 mT, 3 Hz), climbing a 25° slope (8.5 mT, 9 Hz), and advancing over uneven terrain (8.5 mT, 10 Hz) under a uniform rotating magnetic field. Figure 4D shows the reorient–approach–attach–detach loop. Under an 8-mT static horizontal magnetic field, the MSE aligns within seconds. A rotating field (8 mT, 22 Hz) then drives directional locomotion; switching to a static vertical field enables adhesion to the target; finally, reapplying the rotating field triggers detachment. This rapid “locate–adhere–detach” behavior allows the MSE to shuttle freely between various ex vivo cultured tissues and organoids, enabling precise access to target monitoring sites. Besides, bioelectrical signals were recorded with a static vertical field (8 mT) to avoid electromagnetic induction; the rotating field (8 mT, 22 Hz) was used only for navigation before adhesion.

### Electrode performance

**Fig. 5.**
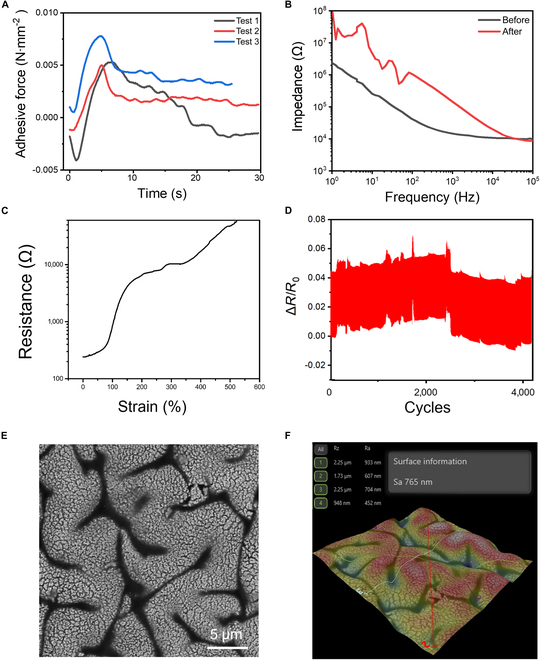
Performance of the electrodes on the MSE. (A) Adhesion force of the MSE on the excised epicardial surface (~3 mN/mm^2^). (B) Impedance changes before and after contact with heart tissue at 1 kHz (initial: ~10 kΩ; post-contact: ~200 kΩ). (C) Resistance change of the 0.5-mm-wide electrodes, demonstrating conductivity under tensile strains up to 160%. (D) Cyclic stretching performance of the electrodes under 40% strain (>4,000 cycles, resistance variation within 5%). (E) SEM image of the gold surface on the MSE. (F) Surface roughness profile of the gold film (Ra ≈ 765 nm, peak-to-valley height ≈ 2.25 μm).

Fig. 5 summarizes the key electromechanical characteristics of the MSE. As shown in Fig. 5A, the MSE achieves an adhesion force of approximately 3 mN/mm^2^ when in direct contact with the epicardial surface of an ex vivo heart, providing a reliable interface for stable attachment and signal collection. Fig. 5B compares the impedance of the MSE before and after contact with the heart surface, measured over a circular sensing area of 1 mm in diameter under 1kHz ac. The impedance measured in air prior to use was approximately 10 kΩ. After one attachment–detachment cycle with cardiac tissue, it increased to ~200 kΩ, possibly due to surface residue, tissue adsorption, or interface alterations. This impedance range is within an acceptable window for continuous ECG signal monitoring.

Figure 5C shows the stretchability of the MSE with a 0.5-mm-wide electrode, which exhibits a maximum conductive strain of about 160% in tensile tests. This strain tolerance fully covers the expected epicardial surface deformation during cardiac cycles, typically ranging from 55% to 70% [45,46]. Although the current open-ended MSE design does not fully leverage this stretchability, it highlights the potential of future closed-loop or encircling designs to achieve more intimate, stable contact with dynamically deforming organs such as the heart [47]. Fig. 5D illustrates the durability of the electrode under cyclic loading, where the device endures over 4,000 stretching cycles at 40% strain with a width of 1 mm. The resistance change remains stable within 5% variation, indicating excellent mechanical and electrical stability under repeated deformation. The surface morphology of the MSE is shown in Fig. 5E and F. SEM imaging and surface profilometry reveal a grooved Au surface with an average roughness of approximately 765 nm and a peak-to-valley height of about 2.25 μm. This microstructured surface contributes to the electrode’s high stretchability and stable electrical performance.

### Overall experimental setup

**Fig. 6.**
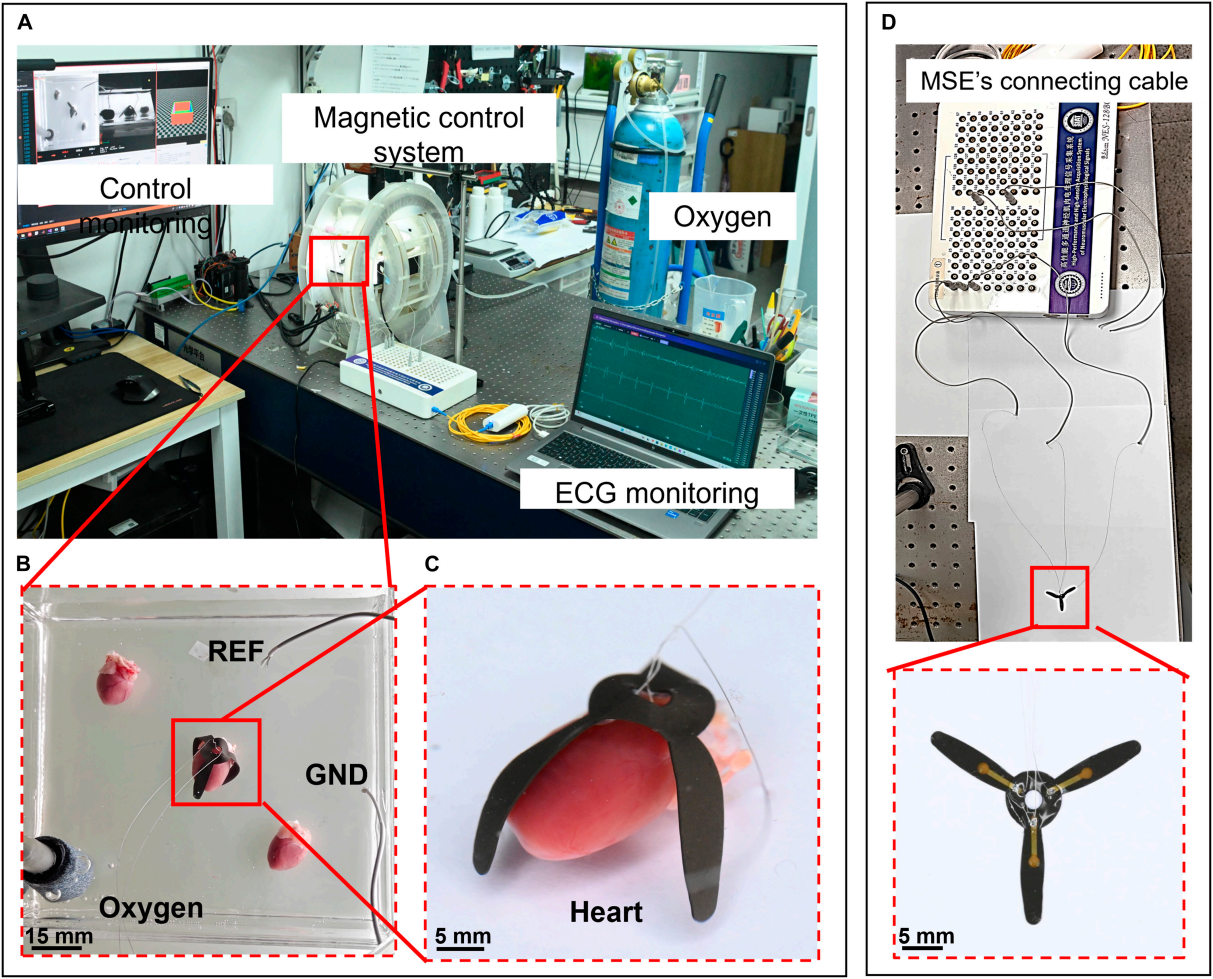
Experimental photographs. (A) Experimental overview. (B) Workspace of MSE in magnetic control system. (C) MSE measuring electrocardiogram (ECG) signals (detailed view). (D) Close-up of MSE’s 3-channel interface.

Fig. 6 presents the overall experimental setup for magnetically actuated ECG signal monitoring using the MSE (Movie S2). As shown in Fig. 6A, the system consists of a magnetic control system, an oxygen supply device, and real-time ECG monitoring equipment. The workspace (Fig. 6B) contains the ex vivo heart model, the MSE, and additional components including the REF electrode, GND electrode, and oxygen diffuser. Fig. 6C provides a detailed view of the MSE conformally attaching to the surface of the heart for ECG signal acquisition (see Movie S3). One electrode channel is clearly in direct contact with the tissue, enabling effective signal recording. Fig. 6D shows a close-up of the MSE’s 3-channel connection to the external data acquisition system. The MSE’s output wires were directly soldered to the device’s connection cables and then inserted into the signal acquisition system.

### Application demonstration: Multi-site ECG detection

**Fig. 7.**
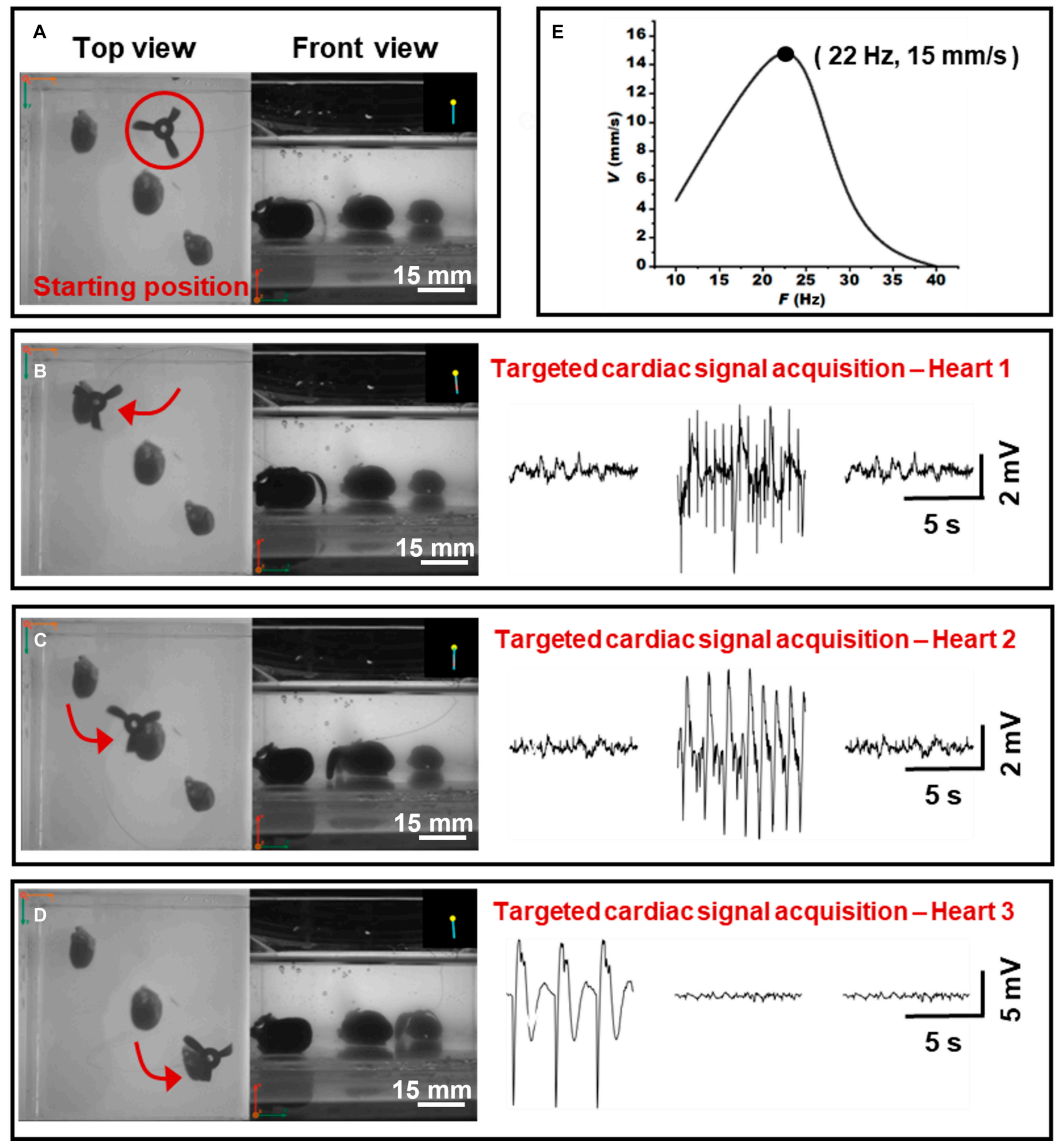
Application demonstration of MSE on controllable ECG recording of ex vivo beating rat hearts. (A to D) The electrode moves sequentially from its initial position to 3 hearts to detect ECG signals and then returns to the starting state. Average signal-to-noise ratio: 28.15 dB. (E) Locomotion speed of the MSE.

To evaluate the operational reliability of MSE in multi-target scenarios, we conducted a continuous cardiac signal monitoring experiment on 3 freshly excised rat hearts (Fig. 7). Movie S4 shows the real-time beating of the hearts and the sequential monitoring process using the MSE. Fig. 7A to D demonstrates the step-by-step navigation of the MSE to each heart surface under a low-frequency rotating magnetic field (*B* = 8 mT, *f* = 2.7 Hz). Upon arrival, the field was switched to a static vertical field (*B* = 8 mT, *f* = 0 Hz) to maintain stable adhesion for signal acquisition. After sampling at 500 Hz and applying a third-order Butterworth bandpass filter (0.5 to 100 Hz), the SNRs of the ECG recordings were calculated as 22.07, 26.03, and 36.34 dB, respectively. The variation in SNR may be attributed to differences in individual heart morphology (e.g., surface curvature and tissue properties). Notably, the third heart achieved an SNR of 36.34 dB, exceeding the clinical diagnostic threshold of 30 dB [48,49]. Only channels with stable low contact impedance were used for analysis; channels without firm contact (e.g., an unattached petal) were excluded and did not affect the recorded trace.

Fig. 7E shows the measured locomotion speed of the MSE, which peaked at 15 mm/s under an 8-mT rotating field at 22 Hz, with a step-out frequency of 40 Hz. For accurate navigation toward each heart, a low-frequency mode (2.7 Hz, ~10.8% of peak frequency) was intentionally selected to reduce mechanical vibration and improve positioning precision, as higher driving frequencies (>10 Hz) tend to degrade docking accuracy due to oscillatory motion.

## Discussion

This study presents an MSE capable of wireless, programmable, and controllable electrophysiological monitoring across multiple tissue targets. With its biomimetic 3-petal design, the MSE achieves controlled locomotion, adaptive adhesion, and autonomous detachment. These capabilities enable a closed-loop “locate–adhere–record–detach” operation under multimodal magnetic fields, supporting reliable, repeatable monitoring on dynamic tissue surfaces such as the beating heart.

In terms of detection performance, the MSE demonstrates excellent mechanical and electrical stability. The Au electrodes remain conductive under strains up to 160%, far exceeding the typical surface deformation range of the heart (55% to 70%). After more than 4,000 stretching cycles, resistance change remains minimal, indicating good reliability of the conductivity. Surface morphology analysis reveals microgrooved patterns on the Au surface, which may enhance both stretchability and stable contact with tissue interfaces.

The system’s functionality was further validated through continuous monitoring on 3 excised beating rat hearts. Using low-frequency rotating fields for precise navigation and static vertical fields for adhesion, the MSE achieved high-fidelity, noninvasive signal acquisition with a peak SNR of 36.34 dB—exceeding the clinical threshold of 30 dB. Its ability to shuttle across multiple targets highlights its potential for repeatable, multi-site electrophysiological detection.

Despite its versatile capabilities, the current MSE system still faces certain limitations. The experiments were conducted within a 3D Helmholtz coil setup, restricting the controllable space to small-scale environments (<10 cm^3^), which limits applicability to larger organs such as isolated rabbit hearts or human-derived organoid assemblies. Future designs may expand the magnetic workspace or explore the parallel control of multiple miniaturized robots within the same field. Another current limitation is that the open 3-petal configuration cannot fully exploit the electrode’s intrinsic stretchability. Nevertheless, the demonstrated strain tolerance (>160%) far exceeds the deformation range of cardiac tissue, ensuring robustness under dynamic motion. More importantly, it opens opportunities for redesigning the MSE into ring-shaped or spherical closed-loop forms, where stretchability will enable conformal wrapping of the heart and more reliable signal acquisition. Additionally, adhesion quality is currently inferred indirectly through impedance changes, which suffer from temporal delay and poor spatial resolution. For instance, localized detachment of a single petal may not be detected in time. Integrating real-time feedback mechanisms could improve precision in positioning and contact control. While the present study primarily focuses on demonstrating electrophysiological monitoring capabilities, future work could build upon recent advances in motion control of magnetically actuated robots [50–52], integrating kinematic modeling and feedback control to further improve targeting precision in complex in vivo environments.

In summary, the MSE integrates magnetic soft robotics with soft electrodes to provide a programmable, multi-site, and minimally invasive platform for electrophysiological signal acquisition on ex vivo organoids, tissue slices, and isolated organs. By overcoming spatial and mechanical limitations of fixed MEAs, it opens new opportunities for dynamic neural circuit mapping, organ-on-chip monitoring, and precision drug screening.

## Ethical Approval

The Animal Ethical Committee of Shenzhen Institute of Advanced Technology, Chinese Academy of Sciences, China (SIAT-IACUC-211014-JCS-HJP-A2068) approved the animal experiments. Three rats were used for ex vivo heart experiments in this study. The laboratory animals’ care followed institutional guidelines of the Chinese Academy of Sciences and the Council for the Purpose of Control and Supervision of Experiments on Animals, Ministry of Public Health, China.

## Data Availability

All data needed to evaluate the conclusions in the paper are present in the paper and/or the Supplementary Materials. Additional data related to this paper may be requested from the authors.
